# Neuroimaging features of primary central nervous system post-transplantation lymphoproliferative disorder following hematopoietic stem cell transplant in patients with β-thalassemia: a case series and review of literature

**DOI:** 10.1186/s13244-024-01605-y

**Published:** 2024-02-14

**Authors:** Xueqing Yang, Xi Deng, Meiqing Wu, Sean W. Chen, Muliang Jiang, Liling Long, Bihong T. Chen

**Affiliations:** 1 Department of Radiology, The First Affiliated Hospital of Guangxi Medical University, No. 6 Shuangyong Road, Nanning, 530021 Guangxi People’s Republic of China; 2 Department of Hematology, The First Affiliated Hospital of Guangxi Medical University, No. 6 Shuangyong Road, Nanning, 530021 Guangxi People’s Republic of China; 3 Department of Medical Oncology & Experimental Therapeutics, City of Hope Comprehensive Cancer Center, 1500 E, Duarte, CA 91010 USA; 4 Department of Diagnostic Radiology, City of Hope Comprehensive Cancer Center, 1500 E, Duarte, CA 91010 USA

**Keywords:** Post-transplantation lymphoproliferative disorder, Central nervous system, β-thalassemia, Hematopoietic stem cell transplantation, Magnetic resonance imaging

## Abstract

**Purpose:**

Primary central nervous system post-transplantation lymphoproliferative disorder (PCNS-PTLD) is a rare but serious complication of hematopoietic stem cell transplantation (HSCT) in patients with severe β-thalassemia. This study aimed to assess the clinical presentation, pathological characteristics, neuroimaging findings, and treatment strategies in patients with β-thalassemia who developed PCNS-PTLD and to compare a case series from our transplant center to reported cases from literature.

**Methods:**

We retrospectively reviewed our hospital database and identified four cases of pathologically confirmed PCNS-PTLD without a history of systemic PTLD in patients with severe β-thalassemia after HSCT. We also performed a relevant literature review on PCNS-PTLD.

**Results:**

The median time from transplantation to diagnosis of PCNS-PTLD was 5.5 months. Intracerebral lesions were usually multiple involving both supratentorial and infratentorial regions with homogeneous or rim enhancement. All patients had pathologically confirmed PCNS-PTLD with three patients having diffuse large B-cell lymphoma and the fourth patient having plasmacytic hyperplasia. There was low response to treatment with a median survival of 83 days.

**Conclusion:**

PCNS-PTLD should be considered in the differential diagnosis of patients with β-thalassemia who had an intracranial lesion on neuroimaging after HSCT.

**Critical relevance statement:**

This case series with a comprehensive review of neuroimaging and clinical characteristics of children with primary central nervous system post-transplantation lymphoproliferative disorder should advance our understanding and improve management of this rare yet severe complication following transplant for β-thalassemia.

**Key points:**

• We assessed clinical presentation, treatment strategies, and neuroimaging characteristics of PCNS-PTLD in patients with β-thalassemia after transplantation.

• Patients with β-thalassemia may have post-transplantation lymphoproliferative disorder presenting as brain lesions on neuroimaging.

• Neuroimaging findings of the brain lesions are helpful for prompt diagnosis and proper management.

**Graphical Abstract:**

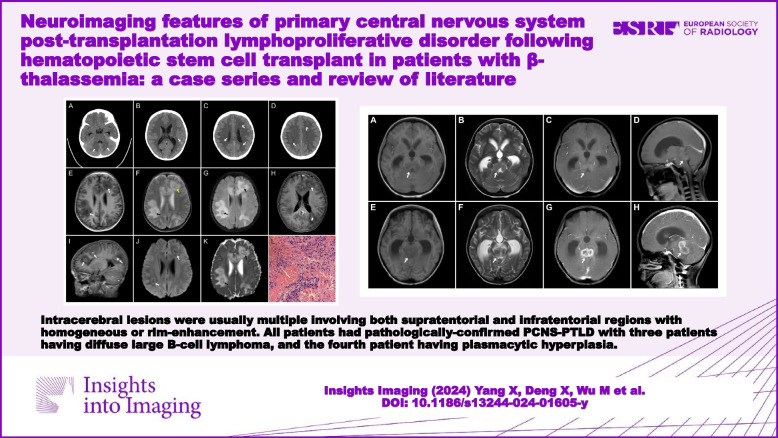

**Supplementary Information:**

The online version contains supplementary material available at 10.1186/s13244-024-01605-y.

## Introduction

Thalassemia is a collection of autosomal recessive disorders categorized by mutations or deletions in genes that regulate hemoglobin leading to impaired globin chain formation, and β-thalassemia is the most common subtype [1]. Hematopoietic stem cell transplantation (HSCT) is a promising treatment for patients with severe β-thalassemia, which may increase the survival rate to 90% [2]. Post-transplantation lymphoproliferative disorder (PTLD) is one of the most severe and challenging complications for patients after HSCT [3,4]. PTLD most commonly involves lymph nodes but may also involve the gastrointestinal tract, lungs, liver, and central nervous system (CNS).

Published literature on primary CNS PTLD (PCNS-PTLD) is limited with isolated case reports or small case series. One study reported more frequent liver and CNS involvement in patients with PTLD after allogeneic hematopoietic stem cell transplantation as compared to solid organ transplantation [5]. Another study reported asymmetrical meningeal thickening without focal brain lesions on brain magnetic resonance imaging (MRI) based on one case of PCNS-PTLD [6]. Several studies have reported neuroimaging features of PCND-PTLD in patients with solid organ transplantation and HSCT, showing neuroimaging features such as multiple supratentorial lesions, with rim-enhancing pattern and peri-lesional edema [7–12]. However, previous studies of few cases were not comprehensive beyond description of PCNS-PTLD. In addition, prior reports did not specifically assess PCNS-PTLD in patients with β-thalassemia after HSCT. More work is needed to understand the underlying pathophysiology to effectively treat this rare but severe complication in patients with thalassemia after HSCT.

Here, we presented a case series of four patients with severe β-thalassemia who developed PCNS-PTLD after HSCT. This report summarized their clinical presentation, pathological characteristics, treatment regimen, and neuroimaging findings. Moreover, we conducted a literature review of the reported PCNS-PTLD cases after HSCT to enhance our understanding of this rare complication.

## Patients and methods

Institutional review board (IRB) of our hospital approved the study (IRB: 2022-E310-01). All cases diagnosed with PCNS-PTLD at our institution from 2013 to 2021 were assessed to identify the relevant cases for patients with β-thalassemia who developed PCNS-PTLD after HSCT. Neuroimaging features assessed for each patient included the following: location, size (maximal diameter), MRI signal/CT density characteristics, hemorrhage, necrosis, edema, enhancement, and the presence or absence of meningeal involvement. The measurement technique and severity criteria for assessing peri-lesional edema are based on the existing literature [13]. Potential risk factors for PCNS-PTLD were investigated including a history of anti-thymocyte globulin therapy and allograft rejection. Additionally, data regarding the clinical variables, neuroimaging features, pathological characteristics, effectiveness of therapy, and survival information of the patients with PCNS-PTLD were assessed.

## Results

### Patient information

Four patients with β-thalassemia and biopsy-proven PCNS-PTLD were identified (Table 1), including one female and three males, ranging in age from 4 to 8 years. Cases were diagnosed at a median age of 6.5 years.

**Table 1:** Demographic and transplant-related information of the four patients in this case series with primary central nervous system post-transplantation lymphoproliferative disorder (PCNS-PTLD) after transplant for β-thalassemia

Characteristic	No./median [range]
**Sex**	
Male	3
Female	1
**Age at diagnosis of PCNS-PTLD, median [range], years**	6.5 [4–8]
**Time from transplantation to PCNS-PTLD, median [range], months**	5.5 [4–11]
**Indications for transplantation**	
Severe β-thalassemia	4
**Type of transplantation**	
Sibling (umbilical cord blood + bone marrow, HLA matched)	2
Unrelated donor (peripheral blood, HLA matched)	1
Sibling (peripheral blood + bone marrow, HLA6/12)	1

### Case series

#### Case 1

**Fig. 1:**
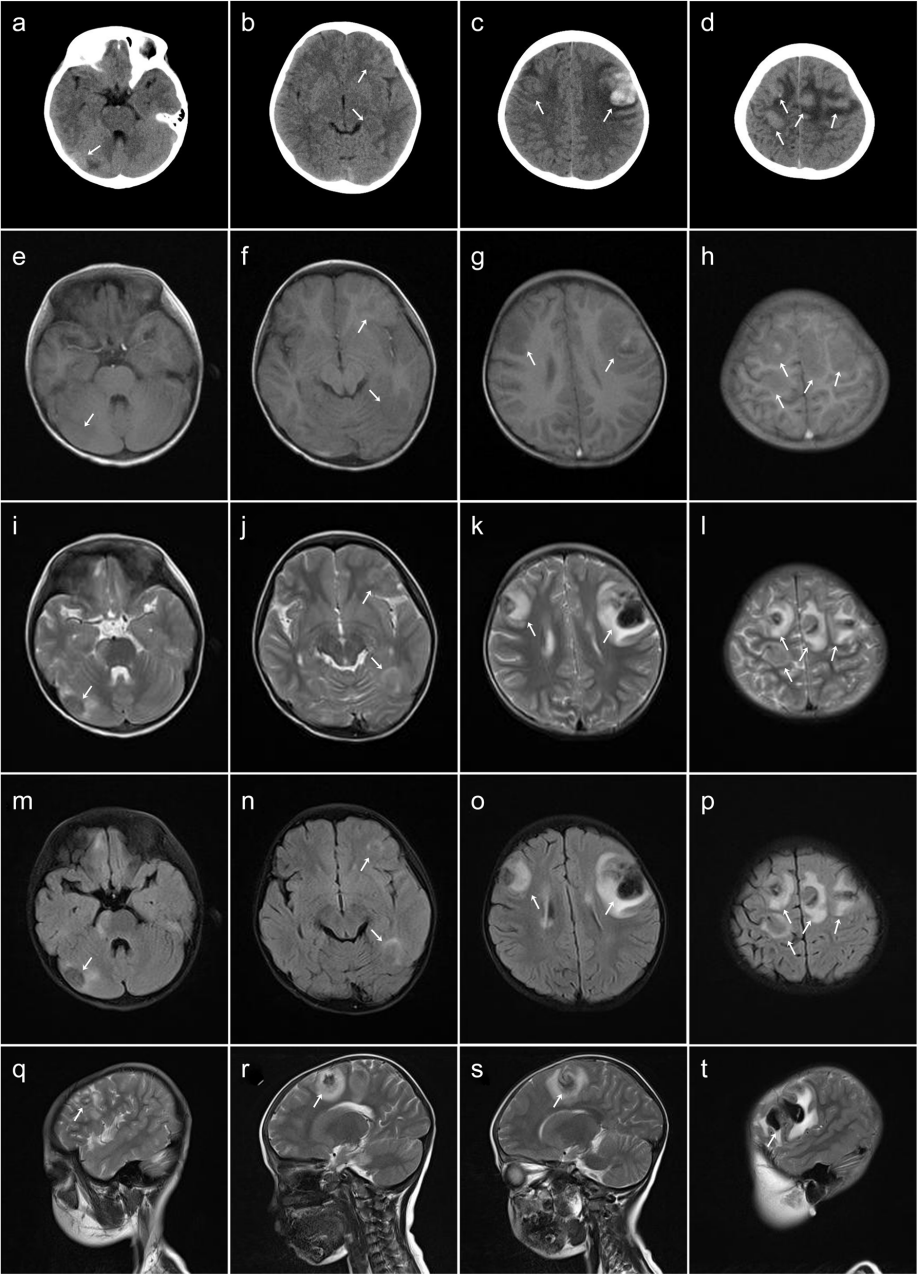
Brain MRI and CT images for the case 1. **a**–**d** Axial CT image showing lesions in the right cerebellar hemisphere, left occipital lobe, pons and frontal-parietal lobes with slight hyperdensity (white arrows), patchy hypodense edema, and hyperdensity. **e**–**h** Axial T1-weighted imaging showing hypointense and heterogeneous hypo- and hyperintense lesions (white arrows) with high signal indicating hemorrhage. **i**–**l** Axial T2-weighted imaging showing extensive peri-lesional edema (white arrows). **m**–**p** Axial T2-fluid-attenuated inversion recovery images showing extensive peri-lesional edema (white arrows). **q**–**t** Sagittal T2-weighted image showing extensive peri-lesional edema (white arrows)

A 5-year-old male patient diagnosed with severe β-thalassemia for more than 4 years was admitted for HSCT. The donor was an unrelated 39-year-old HLA-matched man. Fourteen days after HSCT, the patient developed rash and diarrhea, which was clinically considered as acute graft-versus-host disease (GVHD) (intestinal + cutaneous involvement) and was treated with basiliximab, tacrolimus, methotrexate, and methylprednisolone. Acute GVHD was controlled 127 days after transplantation. The patient developed headache, vomiting, and seizures 145 days after transplantation. The laboratory test of CSF was normal. Routine brain MRI and CT scans (Fig. 1) showed multiple round lesions of various sizes in the right cerebellar hemisphere, left occipital lobe, pontine, frontal, and parietal lobes, with edema around the lesions and hemorrhage within some of the lesions. The lesions in the right cerebellar hemisphere, left occipital lobe, both frontal lobes, parietal lobes, and pons were hypointense on T1-weighted images, heterogeneous hyper- and hypointense on T2-weighted images, and heterogeneous hyper- and hypointense on T2 fluid attenuated inversion recovery images. The parietal and frontal lesions on both sides and left temporal lesion showed heterogeneous signal on T1-weighted images, T2-weighted image, and T2-fluid-attenuated inversion recovery images. On CT, the lesions in the right cerebellar hemisphere, left occipital lobe, pons, and frontal-parietal lobes on both sides showed slight hyperdensity and a band of patchy peri-lesional hypodense edema. There was hyperdensity within the left frontal lobe lesion, which was considered to be associated with hemorrhage.

The patient subsequently underwent emergency craniotomy, and pathologic examination of the surgical specimen suggested possible EBV infection-associated post-transplant lymphoproliferative disorder. The patient was treated with rituximab but died 37 days after the onset of symptoms due to respiratory failure.

#### Case 2

**Fig. 2:**
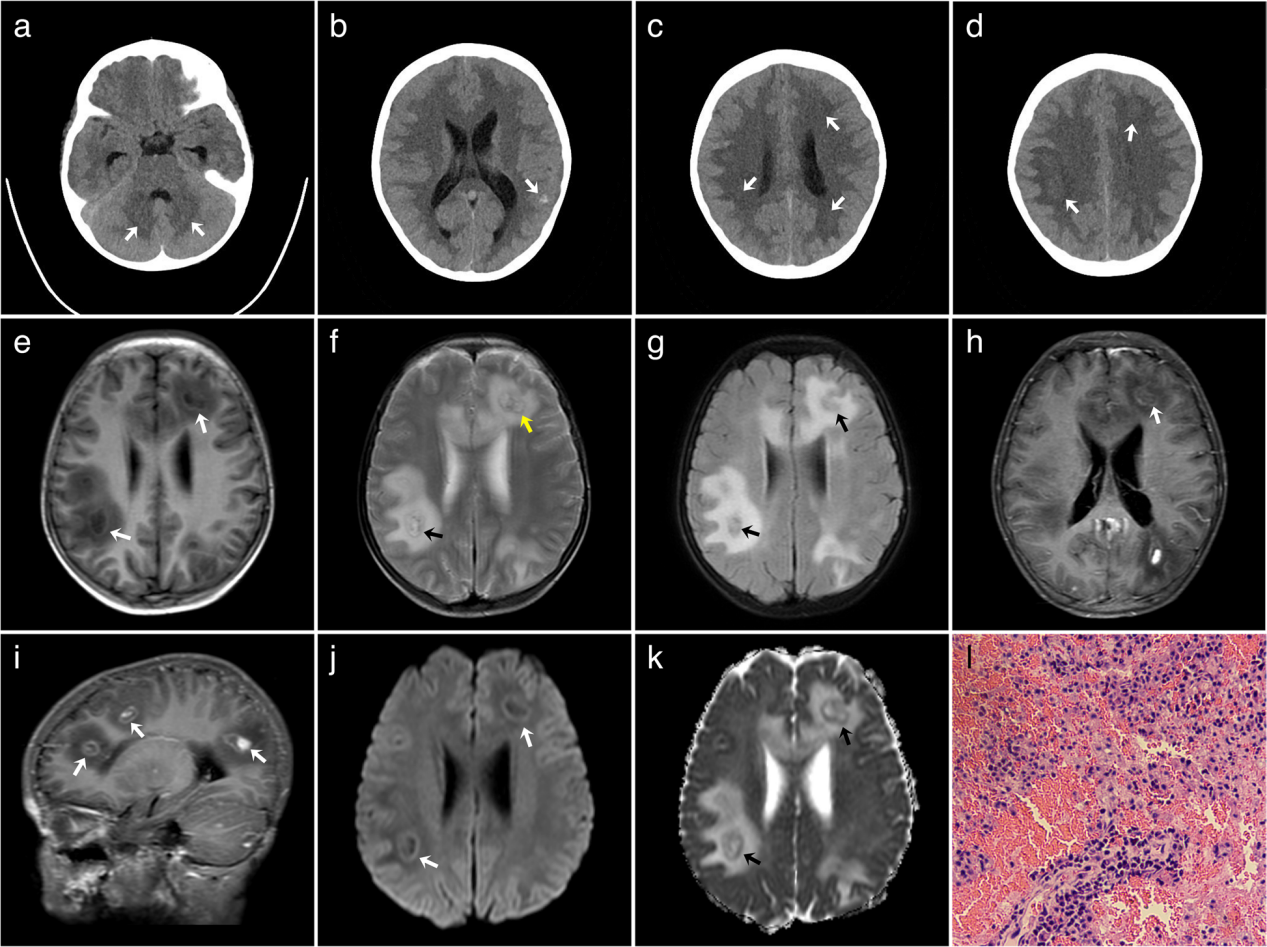
Brain MRI and pathological findings for case 2. Brain CT showing multiple isodensity or hyperdensity lesions (**a**–**d**). Brain MRI with axial-T1-weighted (**e**), T2-weighted (**f**), and fluid-attenuated inversion recovery image (**g**) showing multiple intraparenchymal lesions (white, yellow, and black arrows), contrast-enhanced T1-weighted images (**h**, **i**) showing multiple rim-enhancing lesions (white arrows) in the subcortical white matter, diffusion-weighted image (**j**) and apparent diffusion coefficient image (**k**) showing restricted diffusion of the enhancing rim of the brain lesions (white and black arrows), microscopic presentation of hemorrhage and necrosis accompanied by lymphoid and plasma cell infiltration in a pathology slide (HE × 100) (**l**)

An 8-year-old male patient diagnosed with thalassemia underwent HSCT with a donor who was a sibling with hemizygous HLA. The patient was admitted to the hospital 92 days after HSCT when he developed fever, headache, and vomiting. Emergency laboratory examination showed that EBV RNA was negative, and 4.92 × 10^2^ copies of human *Cytomegalovirus* DNA were detected, and he was treated with cefaclor, sulfamethoxazole, acyclovir, and fluconazole, which was ineffective. Then, he presented with convulsions, loss of speech, and rigidity of the right limb. Brain MRI and CT scans (Fig. 2) revealed multiple nodular lesions in the frontal lobe, parietal lobe, occipital lobe, basal ganglia, cerebellar hemispheres, and cerebellar vermis, and most lesions were isodense on CT. There was patchy hyperdensity in the left occipital lobe lesion, which was considered to be hemorrhage associated with the lesion. On MRI scan, these lesions were hypointense on T1-weighted images, heterogeneous hyper- and hypointense on T2-weighted image, heterogeneous hyper- and hypointense on T2-fluid attenuated inversion recovery images, hyper- and hypointense on diffusion-weighted imaging (DWI), and slightly hyperintense on apparent diffusion coefficient (ADC) imaging. There was slightly hyperintense ring-shaped capsule around some of the larger lesions, and nodular heterogeneous enhancement with edema was also noted. Cerebrospinal fluid next-generation sequencing examination showed the presence of human cytomegalovirus (four sequences detected) and EBV (three sequences detected). Subsequently, the patient underwent craniotomy and pathologic examination identified PTLD as an early lesion, i.e., plasmacytic hyperplasia. After the diagnosis was clarified, rituximab was immediately administered. During the treatment, the child developed a generalized rash, which was acute GVHD (cutaneous form) and was further treated with methylprednisolone and ruxolitinib. The child improved and was discharged after treatment. However, after 235 days of HSCT, the child developed a severe respiratory infection and died of severe pneumonia.

#### Case 3

**Fig. 3:**
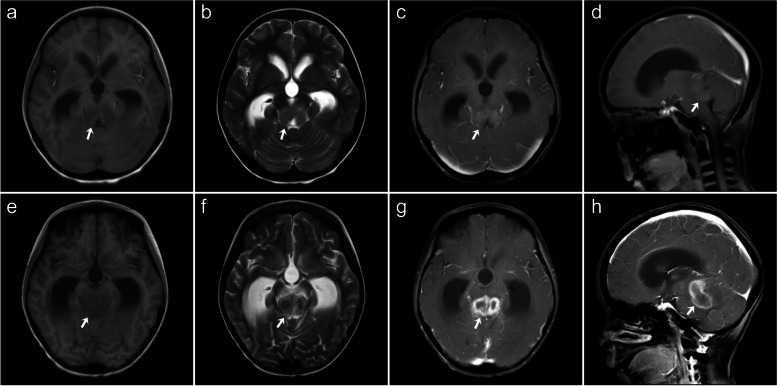
Brain MRI findings for case 3. Brain MRI images (**a**–**d**) at the time of initial diagnosis showing a homogenous lesion (white arrows) being hypointense on T1-weighted imaging (**a**), isointense on T2-weighted imaging (**b**), with mild homogeneous enhancement (**c**, **d**), MRI images (**e**–**h**) obtained 18 days after an initial presentation showing the lesion (white arrows) being enlarged in size (**e**), more heterogeneous in appearance (**f**), and having a new rim-enhancing feature (**g**, **h**)

An 8-year-old female patient diagnosed with thalassemia underwent HSCT with a sibling brother whose donor was fully compatible with HLA 6/6, but the donor was a β-thalassemia gene carrier with genotype βIVS-2-654/βN. The child had recurrent fevers on days 3, 59, and 100 after HSCT, and laboratory tests revealed qualitative positive antibodies to *Mycoplasma pneumoniae* and a quantitative *Mycoplasma pneumoniae* antibody greater than 1:320; EBV DNA count was 2.12 × 10^4^ copies. She was initially treated with cefoperazone sodium, sulbactam sodium, azithromycin, ribavirin, and acyclovir, but the infection was not controlled. She was later treated with meropenem, vancomycin, tigecycline, and fluconazole, and yet her fever persisted. Ultrasound of superficial lymph node showed multiple lymph nodes in the left neck. PET/CT examination showed hypermetabolic lesions in the left neck, bilateral pulmonary hila, mediastinum, para-abdominal aorta, and splenic hilum, concerning for post-transplantation EBV infection-associated lymphoproliferative disease. She was treated with ribavirin and rituximab. Her condition was improved, and she was subsequently discharged. However, the child was readmitted to the hospital on the 114th day of HSCT for lower extremity weakness. Brain MRI (Fig. 3) showed lamellar lesion in the midbrain and pons with blurred borders surrounded by edema in addition to dilatation of the third ventricle, fourth ventricle, and lateral ventricles. The lesion showed hypointense on T1-weighted images, heterogeneous hyper- and hypointense signal on T2-weighted image, and heterogeneous hypointense on T2-fluid-attenuated inversion recovery images and ring-shaped heterogenous enhancement. Methylprednisolone and gammaglobulin were immediately administered, mannitol was used to reduce intracranial pressure, and an intracranial catheter was placed into the ventricle. However, the brain lesions progressed rapidly, and the child’s condition deteriorated. She eventually died of respiratory failure.

#### Case 4

**Fig. 4:**
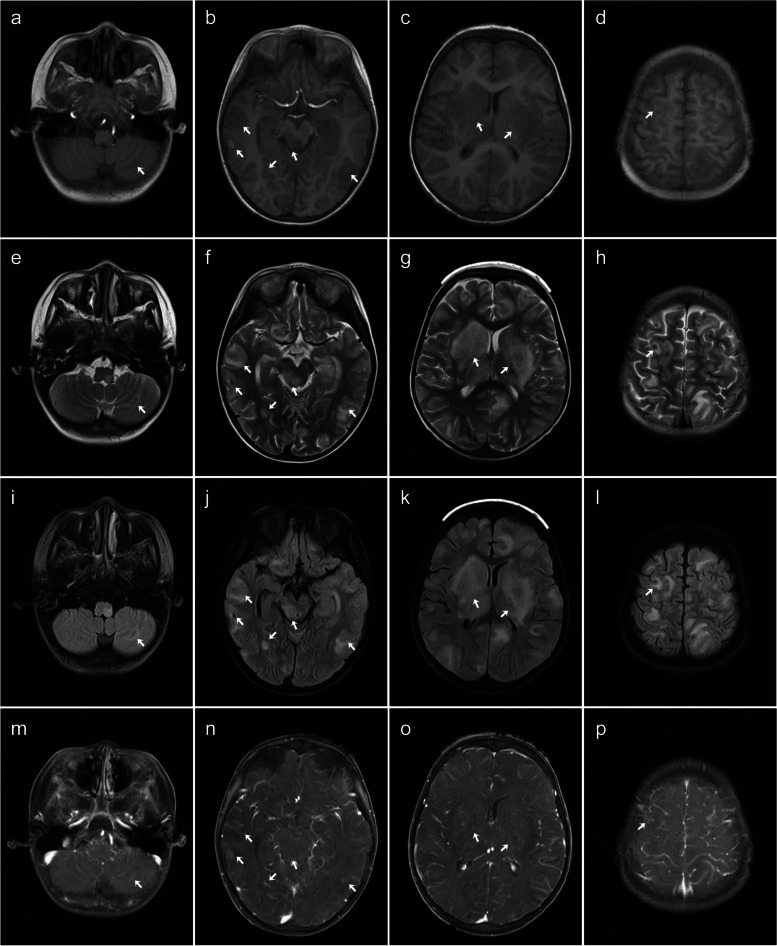
Brain MRI findings for case 4. Brain MRI with axial T1-weighted imaging (**a**–**d**), T2W-weighted imaging (**e**–**h**), and T2-fluid-attenuated inversion recovery image (**i**–**l**) showing multiple intraparenchymal lesions (white arrows) and the T2-weighted image and T2-fluid-attenuated inversion recovery images (**e**–**l**) showing extensive peri-lesional edema (white arrows). The enhance scan (**m**–**p**) showing mild to moderate heterogenous enhancement of the lesions (white arrows)

A 4-year-old male patient diagnosed with major β-thalassemia underwent HSCT. The donor was his younger brother, and their HLA were fully matched. After HSCT, the child had recurrent fever, and the laboratory examination reported the number of CMV-DNA being 7.40 × 10^4^ copies. Antiviral treatment was given with acyclovir, foscarnet, ribavirin, and ganciclovir. On the day 90 after HSCT, the child developed a rash on his trunk, hands, and feet, concerning for chronic GVHD after ruling out the effect of drugs. The rash subsided and improved after treatment with tacrolimus. On the day 128 after HSCT, the child developed convulsions, lethargy, and unresponsiveness. On day 138 after HSCT, the child’s neurological symptoms further worsened, with limb weakness and unresponsiveness. The laboratory tests reported the EBV-DNA quantity being 1.16 × 10^3^ copies/mL. Brain MRI (Fig. 4) showed multiple nodular and flaky lesions on both sides of the cerebral hemispheres, cerebellar hemispheres, pons, and midbrain, with blurred borders, some with hemorrhage and surrounding edema. On brain MRI, the lesions showed hypointensity on T1-weighted images, heterogeneous hyper- and hypointense on T2-weighted image, and heterogeneous hyper- and hypointense on T2-fluid-attenuated inversion recovery images. Some larger lesions showed a slightly hyperintense ring capsule, and the lesions showed nodular light to moderate heterogenous enhancement. On the day 321 after HSCT, enlarged lymph nodes were palpated in the child’s neck, which were surgically removed. The pathology of the neck lymph nodes showed monomorphic B-PTLD-EBV-positive diffuse large B-cell lymphoma (post-germinal center subtype). The patient was subsequently treated with rituximab and methotrexate. However, the patient had poor response to treatment, and his condition deteriorated. His legal guardian requested withdrawal of all treatment and took the patient home for comfort care. The patient was lost to follow-up after leaving the hospital, and his survival status was unknown.

### Summary of neuroimaging features

All patients in this series had multiple brain lesions, with a median size of 2.2 cm and a size range of 0.3–9.8 cm. Most lesions had subcortical white matter distribution and were often lobar in location. The lesions were located in the cerebral lobes, basal ganglia, thalamus, corpus callosum, cerebellum, and brainstem. These lesions appeared to be irregular, flaky, and round-like, among which flaky appearance was the most common. Three patients had bleeding and hemosiderin staining within the brain lesions. These lesions showed iso- or slightly hyperdensity on CT, including small patchy higher density of hemorrhage and lower density of necrosis. The margins of the brain lesions were not delineated on the CT scans. On brain MRI, most lesions were hypointense to isointense on T1-weighted images, heterogeneous hyper- and hypointense on T2-weighted image, and T2-fluid-attenuated inversion recovery images, with mild to moderate heterogenous enhancement. Hemorrhagic components within the lesions were noted as multiple patchy areas of intrinsic hyperintensity on T1-weighted images and hypointensity on T2-weighted image. Some lesions had restricted diffusion of the rim-enhancing component on DWI and slightly hyperintense on ADC map. Edema was identified in all lesions. The lateral ventricles were compressed and deformed in three patients, and the supratentorial ventricles were dilated in one patient.

The clinical characteristics, neuroimaging findings, treatment, and outcome of the four cases reported here are summarized in Table 2, respectively.

**Table 2:** Clinical characteristics, neuroimaging findings, treatment strategies, and outcomes of the four cases with primary central nervous system post-transplantation lymphoproliferative disorder (PCNS-PTLD) following hematopoietic stem cell transplant in patients with *β*-thalassemia from our case series

Characteristic	Case 1	Case 2	Case 3	Case 4
Age, y	5	8	8	4
Sex	M	M	F	M
Brain symptom	Headache, vomiting, seizures	Headache, vomiting, convulsions, loss of speech, and rigidity of his right limb	Appearance of weakness of her lower limbs	Convulsions, lethargy, unresponsiveness, limb weakness, coma
CT	Slight hyperdensity lesions in right cerebellar hemisphere, left occipital lobe, and pontine brain and frontal-parietal, with hyperdensity hemorrhage in the left frontal lobe	Isodensity multiple nodular lesions in the frontal lobe, parietal lobe, occipital lobe, basal ganglia, cerebellar hemispheres, cerebellar vermis, and corpus callosum and patchy hyperdense hemorrhage were found in the left occipital lobe lesion	None	None
MRI	Lesions in right cerebellar hemisphere, left occipital lobe, both frontal lobes, parietal lobes, and pontine lobes were hypointense on T1WI and heterogeneous hyper- and hypointense on T2WI. The parietal and frontal lesions on both sides and left temporal lesion showed heterogeneous signal on T1WI, heterogeneous signal on T2WI, and heterogeneous signal on T2-FLAIR images	The lesions in the frontal lobe, parietal lobe, occipital lobe, basal ganglia, cerebellar hemispheres, cerebellar vermis, and corpus callosum were hypointense on T1WI, heterogeneous hyper- and hypointense on T2WI, heterogeneous hyper- and hypointense on T2-FLAIR, hyper- and hypointense on DWI, and slightly hyperintense on ADC, and slightly hyperintense ring-shaped capsule was around some of the larger lesions, and nodular heterogeneous enhancement on enhancement scan, with scaly edema around them	The lesion in the midbrain and pontine brain was hypointense on T1WI, heterogeneous hyper- and hypointense signal on T2WI, and heterogeneous hypointense on T2-FLAIR and ring-shaped apparent uneven enhancement on the enhancement scan	Lesions in the cerebral hemispheres, cerebellar hemispheres, pons, and midbrain were hypointense on T1WI and heterogeneous hyper- and hypointense on T2WI and T2-FLAIR. Some larger lesions showed a slightly hyperintense ring capsule, and the lesions showed nodular light to moderate uneven enhancement on enhanced scan
CSF				
Protein, mg/L (150.00–450.00)	In normal range	2020.30	316.6	943.80
Glucose, mmol/L (2.80–4.20)	In normal range	2.58	2.75	2.60
Chlorine, mmol/L (120.00–132.00)	In normal range	None	118.5	115.90
NGS	None	CMV was detected with four reads, and EBV was detected with three reads in CSF samples	None	None
Histology	Monomorphic, diffuse large B cell. EBV infection-associated post-transplant lymphoproliferative disorder	Post-transplant lymphoproliferative disorder (early lesion: plasmacytic hyperplasia)	None	Monomorphic B-PTLD-EBV-positive diffuse large B-cell lymphoma (post-germinal center subtype)
Treatment	Craniotomy and medication with rituximab	Craniotomy and medication with rituximab	Medication with methylprednisolone, gammaglobulin, and mannitol. A drainage tube was placed in the ventricle	Craniotomy, and medication with combination of rituximab and MTX
Outcome	Died	Died	Died	Not known

*Abbreviations*: *T1WI* T1-weighted image, *T2WI* T2-weighted image, *T2-FLAIR* T2-fluid-attenuated inversion recovery, *DWI* diffusion-weighted image, *NGS* next-generation sequencing, *CSF* cerebrospinal fluid

## Discussion

This study reported a case series of PCNS-PTLD in pediatric patients with severe β-thalassemia after HSCT. We found that most brain lesions were in a lobar distribution and were located in both supratentorial and infratentorial regions. Most lesions showed homogeneous enhancement initially but progressed to rim enhancement within a short interval and were prone to hemorrhage. Moreover, we reported for the first time the neuroimaging features of an early PCNS-PTLD lesion as a pathological subtype, i.e., plasmacytic hyperplasia, in children with β-thalassemia after HSCT.

PTLD represents a spectrum of disorders, ranging from polyclonal proliferation of early lymphocyte populations to malignant lymphoma, with a high mortality rate of 84.6% [3, 14]. There are a few risk factors for PTLD after HSCT, including pre-transplant T-cell depletion, anti-thymocyte globulin usage, unrelated or HLA-mismatched grafts, acute or chronic GVHD, recipient being EBV seronegative but donor positive, splenectomy, infusion of mesenchymal stem cells, and advanced age at transplantation [15, 16]. Nevertheless, EBV infection and immunosuppression are known as the major causative factors for PTLD [14]. Pediatric patients with primary EBV infection and patients with severe immunosuppression are most at risk for EBV-positive PTLD [14]. Furthermore, PTLD has also been shown to be associated with CMV infection [17]. Our case series was concordant with literature having several identified risk factors such as EBV/CMV infections and immunosuppression. Here, we provided evidence to support the relevance of these reported risk factors for PCNS-PTLD for pediatric patients with severe β-thalassemia.

The present study contributed the first case series of PCNS-PTLD in children with severe β-thalassemia after HSCT. A similar case of PTLD was reported on a child with severe β-thalassemia who developed non-Hodgkin lymphoma (B-cell type) in the nasopharynx after HSCT, but not in the CNS [18]. PTLD after HSCT often develops in the first year after transplantation and is derived from the donor [19]. Rare cases of late-onset PTLD following HSCT are only seen in patients with chronic GVHD who remain immunosuppressed [20]. The median time of 5.5 months from transplantation to PCNS-PTLD diagnosis was noted in our case series, while the median time of 8.4 months was reported in literature (Table S1). The discrepancy in the median time from transplantation to PCNS-PTLD could be partly explained by the difference in the study cohorts. Most of the cases reported in the literature were adults, whereas our cases consisted of children who may have faster cell proliferation and hence the shorter time to develop PTLD than the adult population [21].

There were fewer than 50 PCNS-PTLD cases post-HSCT reported in literature, with neuroimaging data available for only 28 patients [4, 7–10, 22–38], as summarized in our literature review. Patients with PCNS-PTLD commonly presented with symptoms of elevated intracranial pressure such as headache, nausea, vomiting, and decreased consciousness (Table S1) [39, 40]. There have been reports of uncommon findings including inflammatory demyelinating disease and fatal intracranial hemorrhage [34, 37, 38]. In the present case series, patients having PCNS-PTLD after HSCT for β-thalassemia also exhibited similar focal neurological symptoms and increased intracranial pressure, similar to the literature reports.

Neuroimaging is crucial for diagnosing and managing transplant recipients with neurological symptoms, and MRI modality is the most commonly used modality in clinical practice due to its exquisite tissue details. The location for PCNS-PTLD lesions has been variable. In most reported cases, multifocal supratentorial involvement predominates, mostly in a lobar location. The cerebellum and basal ganglia are the second and third most common locations. Lesions are less frequently seen in the brainstem, thalami, and periventricular regions (Tables S1–S2). Lesions are rarely seen in isolation in non-lobar sites such as the infratentorial region or the spinal cord [33]. However, 75% of patients in our case series had both supratentorial and infratentorial involvement and 70% of lesions with subcortical white matter distribution. The discrepancy in brain lesion distribution between our series and the literature could be partially attributed to our focus on pediatric β-thalassemia cases.

There is limited CT density data for PCNS-PTLD lesions. Hyper-, iso-, or hypodensity in brain lesions on CT has been reported [10, 22, 39]. While prior studies reported predominantly hypodense brain lesions, we did not identify such a pattern in the present study. The discrepancy may be due to the difference of brain lesions which may have various underlying disorder for transplantation, duration of hemorrhage within the brain lesions, and extent of necrosis.

The PCNS-PTLD lesions are also variable on brain MRI. Combining literature and our case series, brain lesions could manifest as hemorrhagic, cystic, and necrotic hypercellular masses. Most patients had brain lesions showing hypointense to isointense on T1-weighted sequence [10, 22, 34, 35]. The solid portions of the brain lesions show comparatively low T2-weighted signal intensity due to hypercellularity [22, 25, 36, 39]. The necrotic area was often associated with hemorrhage, especially hemosiderin deposition caused by chronic hemorrhage, so a mixed signal intensity may be seen on T2-weighted sequence. PCNS-PTLD lesions commonly exhibit rim enhancement with vague borders due to central necrosis, similar to the common microscopic finding [7, 39]. Neuroimaging findings in our PTLD case series for β-thalassemia children were comparable to prior studies involving different primary disorders such as leukemia or solid tumors (Table S2) [41, 42].

We also found the size of PCNS-PTLD lesions being unrelated to the presence of hemorrhage, necrosis, or peri-lesional edema. Several small lesions (< 1 cm of longest diameter) had hemorrhage and severe edema in our study. We speculate it might be from injury of blood vessels around the small lesions, predisposing them to hemorrhage and necrosis, resulting in a rim-like enhancing pattern [7, 39]. Additionally, PTLD cells with neovascularity may infiltrate along the perivascular regions, leading to multifocal distribution of PCNS-PTLD lesions.

PCNS-PTLD may progress rapidly in CNS, similar to PTLD occurring in other organs. In the present case series, the lesions were mostly homogeneous in CT density and MRI signal in the early stage. However, within a short duration of time (18 days), these lesions were obviously enlarged, showing heterogeneous CT densities and MRI signals, the enhancement pattern was also changed from solid to rim-enhancement, and the edema was increased significantly (Fig. 3). These imaging findings indicated that PCNS-PTLD progressed rapidly, which may require timely management.

In our case series, only conventional qualitative brain MRI was performed, partly due to the acute presentations of the patients without much time for more complicated imaging and partly due to these cases occurred prior to the more common adaptation of sophisticated imaging techniques. Nevertheless, advanced imaging techniques with both quantitative and qualitative analyses may shed light on the pathophysiology of the PCNS-PTLD and may help to differentiate it from other CNS neoplasia such as glioblastoma, high-grade glioma, and brain metastases [43].

While the literature is limited on advanced imaging of PCNS-PTLD, primary CNS lymphoma has been studied extensively [43]. In addition, two cases from our case series were identified as diffuse large B-cell lymphoma in the brain. Advanced imaging technique such as brain MRI perfusion with dynamic susceptibility contrast (DSC) can be used to assess relative cerebral blood volume (rCBV) as a quantitative parameter for neovascularity in the lymphoproliferative tissue and lymphoma in the brain [44, 45]. Additional brain perfusion technique such as dynamic contrast-enhanced (DCE) MRI may be used to assess vascular permeability of the brain lesions quantitatively.

Susceptibility-weighted imaging (SWI) is sensitive to material that distorts local magnetic field such as blood, calcification, and iron, and the phase data from SWI could be used to measure the intralesional bleeding as seen in our case series [46]. DWI with ADC parameter can be used to measure cellular density and can help to distinguish lymphoproliferative tissue from the extensive peri-lesional edema as in our cases. ADC can be used to distinguish PCNS-PTLD from other CNS neoplasia, and slightly higher ADC values were noted in one of our cases. Magnetic resonance spectroscopy (MRS) has shown elevated peaks for metabolites such as choline, lactic acid, and lipids in lymphoproliferative disorder involving the brain [47]. Lastly, diffusion tensor imaging (DTI) with quantitative parameters such as fractional anisotropy and mean diffusivity can be used to measure the microstructural integrity of white matter fiber bundles and can help to assess the extent of white matter infiltration by the lymphoproliferative tissue in the brain for treatment planning [48].

Diffuse large B-cell lymphoma, a more severe form of PTLD, accounts for most cases both in our series and in literature reports [49, 50]. Similar results were reported in previous studies of PCNS-PTLD following HSCT (Table S1), and most cases were of B-cell origin (Table S3). Plasmacytic hyperplasia is a nondestructive early lesion of PTLD, characterized by polyclonal B-cell growth, normal lymphoid architecture, and the absence of cytogenetic alterations [14]. To the best of our knowledge, there were no prior reports on this early PCNS-PTLD lesion after HSCT for various other primary disorders. Here, we identified novel information on plasmacytic hyperplasia as an early PCNS-PTLD lesion after HSCT in a child with severe β-thalassemia.

There is no consensus on the optimal treatment of PCNS-PTLD after HSCT. It has been treated as a primary CNS lymphoma, and the treatment strategies include chemotherapy most commonly HD-methotrexate ± rituximab [14, 49], rituximab monotherapy [25], immunotherapy [27, 51], and radiotherapy [52]. The intrathecal rituximab injection is promising but with limited success [4]. The emergence of new drugs, such as nivolumab [27] and zanubrutinib [37], may provide additional treatment options when all other therapies are ineffective.

The outcome of patients with PCNS-PTLD is varied. In our series, only two cases reached partial remission, one after treatment with rituximab, prednisone, γ-globulin, and HD-methotrexate and the other after treatment with four cycles of intravenous rituximab. In addition, 75% of our patients survived only 83 days. The median survival of 150 days was reported for patients with PCNS-PTLD after HSCT for diseases other than thalassemia, with 36% of patients were still alive at a median follow-up of 348 days (Tables S3). The shorter survival in the present case series may be related to severe β-thalassemia in our pediatric cohort.

## Conclusion

Patients with severe β-thalassemia who underwent HSCT may develop PCNS-PTLD, an uncommon but severe complication with various clinical and neuroimaging findings. CNS involvement is associated with a poor prognosis. A prompt diagnosis of this complication after HSCT may help to improve the outcome of vulnerable pediatric patients with severe β-thalassemia.

### Supplementary Information

**Additional file 1: Table S1.** 28 patients reported as PCNS-PTLD after hematopoietic stem cell transplantation. **Table S2.** List of Detailed Neuroimaging Findings Collected from All Available Case Reports. **Table S3.** List of detailed clinical data collected from all available case reports

## Data Availability

The data that support the findings of this study are available from the corresponding author upon reasonable request.

## Abbreviations

CMV: *Cytomegalovirus*
CSF: Cerebrospinal fluid
EBV: Epstein-Barr virus
GVHD: Graft-versus-host disease
HLA: Human leukocyte antigen
HSCT: Hematopoietic stem cell transplantation
MTX: Methotrexate
PCNS-PTLD: Primary central nervous system post-transplantation lymphoproliferative disorder
