# Supplementary material for: Neuroimaging features of primary central nervous system post-transplantation lymphoproliferative disorder following hematopoietic stem cell transplant in patients with β-thalassemia: a case series and review of literature
Source: Insights Imaging. 2024 Feb 14;15:40. doi: 10.1186/s13244-024-01605-y (PMC10866827; doi:10.1186/s13244-024-01605-y)
Supplement: Supplementary file 1 — Additional file 1: Table S1. 28 patients reported as PCNS-PTLD after hematopoietic stem cell transplantation. Table S2. List of Detailed Neuroimaging Findings Collected from All Available Case Reports. Table S3. List of detailed clinical data collected from all available case reports [file 13244_2024_1605_MOESM1_ESM.docx]

**Neuroimaging features of primary central nervous system post-transplantation lymphoproliferative disorder following hematopoietic stem cell transplant in patients with β-thalassemia: a case series and review of literature**

**ELECTRONIC SUPPLEMENTARY MATERIAL**

| **Table S1. 28 patients reported as PCNS-PTLD after hematopoietic stem cell transplantation** | |
| --- | --- |
| **Characteristic** | **No. (%)/Median[Range]** |
| **Sex** |  |
| Male | 18 |
| Female | 10 |
| **Median age at transplantation [range], y** | 34.8[2-69] |
| **Median age at diagnosis of PCNS-PTLD [range], y** | 35.5[2.3-70] |
| **Time from transplantation to PCNS-PTLD Median [range], y,( n=28 patients)** | 0.7[0.1-3.2] |
| ≤1 y | 25(89);Median 0.5y |
| ＞1y | 3(11); Median 1.9y |
| **Maintenance immunosuppression,(n=24 patients)** |  |
| Corticosteroids | 11 |
| Cyclosporin | 7 |
| Tacrolimus | 10 |
| Mycophenylate | 9 |
| Methotrexate | 7 |
| ATG | 2 |
| Radiotherapy/Chemotherapy | 5 |
| **Clinical symptoms,( n=26 patients)** |  |
| Intracranial hypertension(headache,nausea,vomiting) | 10(38.5) |
| Mental status change | 7 (26.9) |
| Ataxia | 2 (7.6) |
| Dystonia | 5 (19.2) |
| Consciousness change | 4(15.4) |
| Language disorder | 3(11.5) |
| Vision disorder | 4(15.4) |
| Seizures | 5 (19) |
| Fever | 4(15.4) |
| **EBV status,(n=9 patients)** |  |
| Peripheral blood(+),Cerebrospinal fluid(+) | 2 |
| Peripheral blood(-),Cerebrospinal fluid(+) | 1 |
| Peripheral blood(+) | 6 |
| **CMV status,(n=1 patients)** | 1 |
| **Histopathology,(n=22 patients)** |  |
| M-PTLD | 20 |
| P-PLTD | 2 |
| **Lesions,(n=28 patients)** |  |
| [Undetected](file:///H:\Submission\HCT\table1-8\TABLE%206.%2028%20patients%20reported%20as%20PCNS-PTLD%20after%20hematopoietic%20stem%20cell%20transplantation.xls#RANGE!/javascript:;) | 1(3) |
| Solitary | 10(36) |
| Multiple | 17(61) |
| **Lesion location,(n=27 patients)** |  |
| Supratentorial alone | 21(77.8) |
| Infratentorial alone | 1(3.7) |
| Both supratentorial and infratentorial | 5(18.5) |
| **Specific lesion location,(n=27 patients)** |  |
| Lobar | 23(85.2) |
| Basal ganglia | 3(11.1) |
| choroid plexus | 1(3.7) |
| Thalamic | 2(7.4) |
| Cerebellum | 6(22.2) |
| Brainstem | 2(7.4) |
| Meningeal involvement | 2(7.4) |
| **Enhancement,(n=17 patients)** |  |
| Homogenous | 1(6) |
| Heterogeneous | 3(18) |
| Ring pattern | 13(76) |
| PCNS-PTLD,Primary central nervous system post-transplantation lymphoproliferative disorder; ATG, Antithymocyte globulin; M-PTLD, Monomorphic post-transplantation lymphoproliferative disorder;P-PTLD,Polymorphic post-transplantation lymphoproliferative disorder. | |

| **Table S2. List of Detailed Neuroimaging Findings Collected from All Available Case Reports** | | | | | | | |  |
| --- | --- | --- | --- | --- | --- | --- | --- | --- |
| **Patient [ref]** | **Location** | **Signal** | **Density** | **Enhancement** | **Hemorrhage** | **Necrosis** | **Perifocal edema** |  |
| 1 ^[7]^ | Singular lesion;parietal lobe | - | - | Ring pattern | - | - | Yes |  |
|  |  |  |  |  |  |  |  |  |
| 2 ^[8]^ | Multiple lesions;cerebellum,periventricular | T2/FLAIR, ↑ | ↓ | Ring pattern | Yes | Yes | Yes |  |
| 3 ^[9]^ | UD | UD | UD | UD | UD | UD | UD |  |
|  |  |  |  |  |  |  |  |  |
| 4 ^[10]^ | Multiple lesions;temporal, occipital lobes | - | ↑ | - | Yes | - | Yes |  |
| 5 ^[11]^ | Singular lesion;frontal lobe | - | - | Ring pattern | Yes | - | Yes |  |
| 6^[12]^ | Multiple lesions;bilateral cerebral hemispheres | - | - | Ring pattern | - | - | Yes |  |
| 7 ^[13]^ | Multiple lesions;frontal, occipital lobes | FLAIR,↑ | - | Ring pattern | - | - | Yes |  |
|  |  |  |  |  |  |  |  |  |
| 8^[14]^ | Singular lesion;frontal lobe | - | - | Ring pattern | - | - | Yes |  |
| 9 ^[15]^ | Multiple lesions;frontal lobe,cerebellum | T2,Iso | - | - | - | - | Yes |  |
| 10^[16]^ | Multiple lesions;lobe,basal ganglia,cerebellum,brainstem | - | - | Ring pattern | - | - | Yes |  |
| 11^[17]^ | Multiple lesions;bilateral cerebral hemispheres | - | - | Ring pattern | - | - | Yes |  |
| 12^[18]^ | Multiple lesions;bilateral cerebral hemispheres | - | - | Ring pattern | yes | - | - |  |
| 13 ^[18]^ | Singular lesion;temporal lobe | - | - | Heterogeneous contrast | - | - | - |  |
|  |  |  |  |  |  |  |  |  |
| 14^[18]^ | Multiple lesions;bilateral frontal lobes | - | - | Ring pattern | - | - | - |  |
| 15^[19]^ | Multiple lesions;bilateral choroid plexus | - | - | Heterogeneous contrast | - | - | - |  |
|  |  |  |  |  |  |  |  |  |
| 16^[20]^ | Singular lesion;parietal lobe | T2,↓ | - | - | - | - | Yes |  |
| 17^[21]^ | Singular lesion;the junction between the pons and the right cerebellar peduncle | - | - | - | - | - | - |  |
| 18^[22]^ | Multiple lesions;bilateral deep gray matter nuclei,cerebellum | - | - | Ring pattern | - | - | Yes |  |
| 19^[22]^ | Singular lesion;basal ganglia | - | - | Ring pattern | - | - | Yes |  |
| 20^[23]^ | Singular lesion;parietal lobe | - | - | Ring pattern | - | - | Yes |  |
| 21^[24]^ | Multiple lesions;parietal,occipital lobes,cerebellum | - | - | Incomplete ring pattern | yes | - | Yes |  |
| 22^[25]^ | Multiple lesions;bilateral cerebral hemispheres,basal ganglia | FLAIR,↑ | - | - | - | - | Yes |  |
| 23^[25]^ | Multiple lesions | ↑ | - | - | - | - | - |  |
| 24^[26]^ | Singular lesion;thalamus | ↓ | - | Ring pattern | - | - | Yes |  |
| 25^[27]^ | Multiple lesions;forebrain white matter | - | - | - | - | - | - |  |
| 26^[27]^ | Multiple lesions;parietal, temporal lobes | - | - | - | - | - | - |  |
| 27^[27]^ | Multiple lesions;cerebral hemispheres, pons | - | - | - | - | - | - |  |
| 28^[28]^ | Multiple lesions;supra-and infra-tentorium | - | - | - | - | - | - |  |
| ↓, hypointense; Iso, isontense; ↑, hyperintense; -, not mentioned; UD,undetected. | | | | | | | |  |

| **Table S3. List of detailed clinical data collected from all available case reports** | | | | | | | | | | | |
| --- | --- | --- | --- | --- | --- | --- | --- | --- | --- | --- | --- |
| **Patient [ref]** | **Gender/age (y)** | **Diag-nosis** | **Transplant type** | **Conditioning regimen** | **EBV^a^ status** | **Clinical presentation** | **Duration^b^ (days)** | **Histology Subtype** | **Treatment** | **Overall Survival, d^c^** | **Outcome** |
| 1 ^[7]^ | M/17 | ALL | MUD | TC, MMF | - | C,S | 621 | M-PTLD | RI,RIX,RT | 397 | Died of PTLD and MODS |
| 2 ^[8]^ | M/49 | SAML | - | CSA,TC, MMF,CS | - | C,A,D | 1169 | M-PTLD (DLBCL) | RIX,DLI | 56 | Died of PTLD |
| 3 ^[9]^ | M/53 | MM | ASCT | Not used | PB | C, F,LY | 270 | M-PTLD | MTX,RIX,RT | - | CR |
|  |  |  |  |  | (+), CSF |  |  | (T-cell ) |  |  |  |
|  |  |  |  |  | (+) |  |  |  |  |  |  |
| 4 ^[10]^ | M/58 | MDS | MUD | CT,RT,MMF,CS,MTX | - | H | 340 | M-PTLD | - | 8 | Died |
| 5 ^[11]^ | M/51 | MDS | HT | TC,CS | - | UM | 306 | M-PTLD (DLBCL) | RIX,DLI | 331 | CR |
| 6^[12]^ | M/29 | AML | UCBT,HT | TC,CS,MMF | - | S | 172 | M-PTLD | RIX,RT | 72 | PR |
|  |  |  |  |  |  |  |  |  |  |  | (Died of IIP) |
| 7 ^[13]^ | F/27 | ALL | MUD | CSA,MTX, | - | H,D | 173 | M-PTLD (DLBCL) | RI,RIX | 180 | CR |
|  |  |  |  | MMF |  |  |  |  |  |  |  |
| 8^[14]^ | M/59 | FL | - | CSA,CS | - | S | 90 | M-PTLD (DLBCL) | CT,RT | - | Died of PTLD |
| 9 ^[15]^ | F/11 | BCR-ABL-CML | UCBT,HT | MMF | - | N,VO,D | 82 | M-PTLD (DLBCL) | CTL,RIX, HD-MTX, CYT,NIV | 365 | CR |
| 10^[16]^ | M/49 | CML | MUD | TC,CS,MMF | - | F,C,A | 108 | M-PTLD | RI,RT,CS | 47 | Died of sepsis |
| 11^[17]^ | F/27 | ALL | MUD | TC,MTX | - | N,D | 288 | M-PTLD (DLBCL) | RT | 17 | Died of PTLD |
| 12^[18]^ | F/27 | MF | HT | - | - | H | 120 | - | RI,MTX, DLI,RT | 120 | Died of PTLD and ^pneumon-ia^ |
| 13 ^[18]^ | F/50 | MM | MUD | - | PB | H,LA | 267 | - | RI,RT | 267 | CR |
|  |  |  |  |  | (+), |  |  |  |  |  |  |
|  |  |  |  |  | CSF |  |  |  |  |  |  |
|  |  |  |  |  | (+) |  |  |  |  |  |  |
| 14^[18]^ | M/46 | MM | MSD | - | PB | LA | 309 | - | RI,RIX,RT,DLI | 309 | Died of PTLD |
|  |  |  |  |  | (-), CSF |  |  |  |  |  |  |
|  |  |  |  |  | (+) |  |  |  |  |  |  |
| 15^[19]^ | F/30 | AA | - | TC,CS | PB | H,N,VO | 230 | M-PTLD | HD-MTX,RIX | 857 | CR |
|  |  |  |  |  | (-), CSF |  |  |  |  |  |  |
|  |  |  |  |  | (-) |  |  |  |  |  |  |
| 16^[20]^ | M/2 | AN | ASCT | Not used | - | - | 99 | M-PTLD (DLBCL) | TR | 996 | CR |
| 17^[21]^ | F/16 | AA | PB-SCT | CSA,MTX | CMV (+), BKV (+) | H,N,VO,D,VE | 60 | M-PTLD | RIX | - | CR |
| 18^[22]^ | M/4 | JML | UCBT | CS,RIX | PB(+) | T,W | 210 | M-PTLD | IT-RIX,MTX,HYD | 510 | CR |
| 19^[22]^ | M/4 | XLP | MUD | Alem | - | B,S,L,CO | 42 | M-PTLD (DLBCL ) | IT-MTX/HYD,RI,CTL,IT-RIX | 462 | CR |
| 20^[23]^ | F/31 | CML | BMT | TC,MTX | - | F,UM | 200 | P-PLTD | RI,DLI | 45 | Died of CML progressi-on |
| 21^[24]^ | M/38 | CML | BMT | TC,CS | - | S | 196 | P-PLTD | RT,CS | 12 | Died of PTLD |
| 22^[25]^ | M/69 | MDS | MUD | CT,RT,Alem | PB(+) | CG,W | 365 | M-PTLD | RI,RIX | 98 | PR(Died ^of leukemia^) |
| 23^[25]^ | M/63 | AML | HT | - | PB(+) | CG | 210 | M-PTLD | RIX | 120 | CR |
| 24^[26]^ | M/39 | BCR-ABL-ALL | HT | MMF,CSA,MTX | - | H,UM,D | 203 | M-PTLD (DLBCL ) | RI,RIX,MTX,RT | 194 | CR |
| 25^[27]^ | M/51 | AML,APL | PB-SCT | CYT,DNR | - | - | 90 | - | CS,CSA | 30 | Died of PTLD |
| 26^[27]^ | M/51 | MM | PB-SCT | MMF,CS,M | - | - | 330 | - | CS,MMF | 7 | Died of PTLD |
| 27^[27]^ | M/6 | LL | BMT | CS,CT | - | - | 690 | - | - | 660 | Died of PTLD |
| 28^[28]^ | F/20 | ALL | - | CT,RT,MTX,CSA,ATG | PB(+) | F,H,N,VO,CG,D | 49 | M-PTLD (DLBCL ) | IT-RIX/DEX | - | CR |
|  |  |  |  |  |  |  |  |  |  |  |  |
| F,Female; M,Male;ASCT,Autologous stem cell transplantation; HT,Haploidentical transplantation; UCBT,Umbilical cord blood transplantation; HLA,Human leukocyte antigen; MUD,Matched unrelated donor; MSD,Matched sibling donor; PB-SCT,Peripheral blood stem cell transplantation; BMT,Bone marrow transplantation; EBV,Epstein-barr virus; M-PTLD, Monomorphic post-transplantation lymphoproliferative disorder; P-PTLD,Polymorphic post-transplantation lymphoproliferati- ve disorder; DLBCL,Diffuse large B cell lymphomas; ALL,Acute lymphoblastic leukemia; SAML,Secondary acute myelogenous leukemia; MM,Multiple myeloma; MDS,Myelodysplastic syndrome; FL,Follicular lymphoma; CML,Chronic myeloid leukemia; MF,Myelofibrosis; AA,Aplastic anemia; AN,Adrenal neuroblastoma; JML,Juvenile myelomonocytic leukaemia; XLP,X linked lymphoproliferative disease; APL,Acute promyelocytic leukemia; LL,Lymphoblastic leukemia; TC:Tacrolimus; MMF,Mycophenolate mofetil; CSA,Cyclosporine A; MTX,Methotrexate; Alem,Alemtuzumab; CYT,Cytarabine; DNR,Daunorubicin; M,Melphalan; ATG,Antithymocyte globulin; RI,Reduction of Immunosuppression; RIX,Rituximab; CS,Corticosteroids; CT,Chemotherapy; RT,Radiotherapy; DLI,Donor Lymphocyte Infusion; HD-MTX,High-Dose-Methotrexate; NIV,Nivolumab; HYD,Hydrocortisone; CTL,Cytotoxic T-lymphocyte; IT-MTX,Intrathecal Methotrexate; TR,Total Resection; IT-RIX,Intrathecal Rituximab; DEX,Dexamethasone; EBV,Epstein-barr virus; CMV,Cytomegalovirus; BKV,Polyomavirus; CSF,Cerebrospinal fluid; C,Confusion; S,Seizures; A,Ataxia; D,Diplopia; F,Fever; LY,Lymphadenectasis; H,Headache; UM,Unilateral motor weakness; N,Nausea; VO,Vomiting; LA,Lalopathy; VE,Vertigo; T,Tremor;W,Trouble walking; B,Behavioral; L,Language; CO,Coma; CG,Cognitive; MODS,Multiple Organ Dysfunction Syndrome; CR,Complete Response; PR,Partial Response; IIP,Idiopathic Interstitial Pneumonia. | | | | | | | | | | | |
| ^aOne patient had reactivation of CMV and BKV.^ | | | | | | | | | | | |
| ^bTime from HSCT until the development of CNS-PTLD.^ | | | | | | | | | | | |
| ^cOutcomes measured from date of PCNS-PTLD diagnosis.^ | | | | | | | | | | | |

**Literature search methods**

Literature was reviewed by searching multiple databases using the following terms: “thalassemia”, “lymphoproliferative disorders”, “PTLD”, “hematopoietic stem cell transplantation”, “HSCT”, “transplantation”, “primary”, “cerebri”, “cerebral”, “brain”, “central nervous system”, and “nervous system” joined in logical combinations. Citations of relevant studies were checked for relevant articles. Only articles in English were retrieved and reviewed.

**Literature search results**

Literature search identified 47 patients from 30 reports that fulfilled the definition of PCNS-PTLD after HSCT. Of them, 19 patients were eliminated for incomplete data. A total of 28 patients from the literature were identified including 18 males and 10 females with underlying disorders including leukemia, lymphoma, myeloma, etc., and their information is presented in the Supplementary Material (**Tables S1–S3)**^1–22^. Although our literature search also identified few cases with β-thalassemia who developed PCNS-PTLD, the detailed neuroimaging features of these patients were not available from these reports and therefore these cases were not included in **Tables S1-S3**. **Table S1** lists the abstracted information on the patients from literature including patient demographics, immunosuppression, clinical symptoms, histopathology, and neuroimaging data. The median time from HSCT to PCNS-PTLD diagnosis for all cases from literature was 0.7 years (range: 0.1–3.2 years).

The most common symptoms for the patients from literature were the symptoms related to intracranial hypertension such as headache, nausea and vomiting in 10 patients and mental status changes in seven patients, followed by seizures and dystonia in five patients respectively. Several patients had symptoms such as changes in consciousness, language disorder, visual disturbance and fever. Ataxia was uncommon, with only two occurrences observed throughout the illness.

Among all patients from literature, multiple lesions were noted in 17 patients and a solitary lesion pattern was noted in 10 patients. Supratentorial lesions were noted in 77.7% of the patients, and usually in a lobar distribution. All brain lesions in the 17 patients who received contrast administration displayed some enhancement. A total of 13 patients had at least one lesion with rim enhancement, one with homogenous enhancement and three with heterogeneous enhancement. Five cases showed evidence of bleeding within the brain lesions. **Table S2** details the neuroimaging findings from all available case reports in the literature.

Among the 28 patients from literature, monomorphic diffuse large B-cell lymphoma was the most prevalent histological subtype of PTLD, with 10 patients belonged to the M-PTLD subtype. The remaining patients had various PTLD subtypes including one patient with T-cell lymphoma, two patients with polymorphic PTLD (P-PTLD) subtype, fifteen patients with unknown subtypes (**Table S3**).

The patients identified from literature underwent various treatments (**Table S3**). Most patients were treated initially with reduction of immunosuppression and 17 patients received at least one intravenous infusion of rituximab. Whole-brain irradiation was performed in 11 patients. Seven patients received at least one cycle of HD- methotrexate (3.5g/m^2^). Some patients also received systemic chemotherapy, nivolumab, donor lymphocyte infusion, EBV-specific cytotoxic T lymphocyte infusion, corticosteroids, and antiviral therapy alone or in combination. The median follow-up time was 150 days (7–996 days). At the last follow-up evaluation, 13 participants were still alive at the time of the reports, and they had a full recovery with resolution of brain lesions on the subsequent brain MRI scans.

Fifteen patients died, with 10 patients due to advanced PCNS-PTLD, two from primary disease progression or recurrence, two from idiopathic interstitial pneumonia and sepsis, and one from unknown cause.

1. Castellano-Sanchez AA, Li S, Qian J, Lagoo A, Weir E, Brat DJ. Primary Central Nervous System Posttransplant Lymphoproliferative Disorders. *Am J Clin Pathol*. 2004;121(2):246-253. doi:10.1309/N82CTQ1J0XEVEFQB

2. Sakamoto H, Itonaga H, Taguchi J, et al. Central nervous system post-transplant lymphoproliferative disorder after allogeneic hematopoietic stem cell transplantation: The Nagasaki transplant group experience. *Leukemia Research Reports*. 2019;11:27-30. doi:10.1016/j.lrr.2019.04.003

3. Ishikawa T, Shimizu H, Takei T, et al. Monomorphic post-transplant T-lymphoproliferative disorder after autologous stem cell transplantation for multiple myeloma. *[Rinsho Ketsueki] The Japanese Journal of Clinical Hematology*. 2016;57(1):36-40.

4. Aisa Y, Mori T, Nakazato T, et al. Primary central nervous system post-transplant lymphoproliferative disorder presenting as cerebral hemorrhage after unrelated bone marrow transplantation. *Transplant Infectious Disease*. 2009;11(5):438-441. doi:10.1111/j.1399-3062.2009.00409.x

5. Kittan NA, Beier F, Kurz K, et al. Isolated cerebral manifestation of Epstein-Barr virus-associated post-transplant lymphoproliferative disorder after allogeneic hematopoietic stem cell transplantation: a case of clinical and diagnostic challenges: PTLD vs. encephalitis after alloHSCT. *Transplant Infectious Disease*. 2011;13(5):524-530. doi:10.1111/j.1399-3062.2011.00621.x

6. Mayumi A, Yamashita T, Matsuda I, et al. Toxoplasmic Encephalitis Followed by Primary EBV-Associated Post-Transplant Lymphoproliferative Disorder of the Central Nervous System in a Patient Undergoing Allogeneic Hematopoietic Stem Cell Transplant: A Case Report. *Transplantation Proceedings*. 2020;52(9):2858-2860. doi:10.1016/j.transproceed.2020.08.002

7. Shimizu H, Saitoh T, Koya H, et al. Discrepancy in EBV-DNA load between peripheral blood and cerebrospinal fluid in a patient with isolated CNS post-transplant lymphoproliferative disorder. *Int J Hematol*. 2011;94(5):495-498. doi:10.1007/s12185-011-0951-3

8. Lieberman F, Yazbeck V, Raptis A, Felgar R, Boyiadzis M. Primary central nervous system post-transplant lymphoproliferative disorders following allogeneic hematopoietic stem cell transplantation. *J Neurooncol*. 2012;107(2):225-232. doi:10.1007/s11060-011-0739-6

9. Tang TC, Chuang WY, Chang H. Isolated cerebral post-transplant lymphoproliferative disorder in a lymphoma recipient. *J Can Res Ther*. 2013;9(3):534. doi:10.4103/0973-1482.119373

10. Kassa C, Reményi P, Sinkó J, Kállay K, Kertész G, Kriván G. Successful nivolumab therapy in an allogeneic stem cell transplant child with post-transplant lymphoproliferative disorder. *Pediatr Transplant*. 2018;22(8):e13302. doi:10.1111/petr.13302

11. Hamadani M, Martin LK, Benson DM, Copelan EA, Devine SM, Hofmeister CC. Central nervous system post-transplant lymphoproliferative disorder despite negative serum and spinal fluid Epstein–Barr virus DNA PCR. *Bone Marrow Transplant*. 2007;39(4):249-251. doi:10.1038/sj.bmt.1705575

12. Azuma Y, Nakaya A, Fujita S, et al. [Primary Central Nervous System Post-Transplant Lymphoproliferative Disorder in a Patient with Acute Lymphocytic Leukemia]. *Gan To Kagaku Ryoho*. 2015;42(8):1005-1007.

13. Balaguer-Rosello A, Piñana JL, Bataller L, et al. Central Nervous System Involvement in Epstein–Barr Virus-Related Post-Transplant Lymphoproliferative Disorders after Allogeneic Hematopoietic Stem Cell Transplantation. *Transplantation and Cellular Therapy*. 2021;27(3):261.e1-261.e7. doi:10.1016/j.jtct.2020.12.019

14. Inoue H, Rai S, Tanaka H, et al. Epstein–Barr Virus-Induced Post-Transplant Lymphoproliferative Disorder of the Central Nervous System Successfully Treated with Chemo-Immunotherapy. *Viruses*. 2020;12(4):416. doi:10.3390/v12040416

15. Sano H, Fujimoto M, Okuno K, et al. Epstein–Barr virus-associated posttransplant lymphoproliferative disorder involving the central nervous system following autologous hematopoietic stem cell transplantation for neuroblastoma. *SpringerPlus*. 2014;3(1):582. doi:10.1186/2193-1801-3-582

16. Januszkiewicz-Lewandowska D. Utility of quantitative EBV DNA measurements in cerebrospinal fluid for diagnosis and monitoring of treatment of central nervous system EBV-associated post-transplant lymphoproliferative disorder after allogenic hematopoietic stem cell transplantation. *Ann Transplant*. 2014;19:253-256. doi:10.12659/AOT.890372

17. Bonney DK, Htwe EE, Turner A, et al. Sustained response to intrathecal rituximab in EBV associated Post-transplant lymphoproliferative disease confined to the central nervous system following haematopoietic stem cell transplant: Intrathecal Rituximab in CNS PTLD. *Pediatr Blood Cancer*. 2012;58(3):459-461. doi:10.1002/pbc.23134

18. Nagafuji K, Eto T, Hayashi S, et al. Donor lymphocyte transfusion for the treatment of Epstein–Barr virus-associated lymphoproliferative disorder of the brain. *Bone Marrow Transplant*. 1998;21(11):1155-1158. doi:10.1038/sj.bmt.1701205

19. Terasawa T, Ohashi H, Tsushita K, et al. Failure to Detect Epstein-Barr Virus (EBV) DNA in Plasma by Real-Time PCR in a Case of EBV-Associated Posttransplantation Lymphoproliferative Disorder Confined to the Central Nervous System. *Int J Hematol*. 2002;75(4):416-420. doi:10.1007/BF02982135

20. Kordelas L, Trenschel R, Koldehoff M, Elmaagacli A, Beelen DW. Successful Treatment of EBV PTLD with CNS Lymphomas with the Monoclonal Anti-CD20 Antibody Rituximab. *Oncol Res Treat*. 2008;31(12):691-693. doi:10.1159/000165057

21. Yang TT, Chen WH, Zhao YM, Fu HR, Huang H, Shi JM. Zanubrutinib Treatment of Central Nervous System Posttransplant Lymphoproliferative Disorder After Allogeneic Hematopoietic Stem Cell Transplantation: A Case Report. *Front Oncol*. 2021;11:672052. doi:10.3389/fonc.2021.672052

22. Liu L, Liu Q, Feng S. Management of Epstein–Barr virus-related post-transplant lymphoproliferative disorder after allogeneic hematopoietic stem cell transplantation. *Therapeutic Advances in Hematology*. 2020;11:204062072091096. doi:10.1177/2040620720910964
